# Widely cited global irrigation statistics lack empirical support

**DOI:** 10.1093/pnasnexus/pgaf323

**Published:** 2025-11-11

**Authors:** Arnald Puy, Seth N Linga, Nanxin Wei, Samuel Flinders, Bethan Callow, Grace Allen, Beatrice Cross, Carmen Aguiló-Rivera, Bruce Lankford

**Affiliations:** School of Geography, Earth and Environmental Sciences, University of Birmingham, Birmingham B15 2TT, United Kingdom; School of Geography, Earth and Environmental Sciences, University of Birmingham, Birmingham B15 2TT, United Kingdom; School of Geography, Earth and Environmental Sciences, University of Birmingham, Birmingham B15 2TT, United Kingdom; School of Geography, Earth and Environmental Sciences, University of Birmingham, Birmingham B15 2TT, United Kingdom; School of Geography, Earth and Environmental Sciences, University of Birmingham, Birmingham B15 2TT, United Kingdom; School of Geography, Earth and Environmental Sciences, University of Birmingham, Birmingham B15 2TT, United Kingdom; School of Geography, Earth and Environmental Sciences, University of Birmingham, Birmingham B15 2TT, United Kingdom; School of Geography, Earth and Environmental Sciences, University of Birmingham, Birmingham B15 2TT, United Kingdom; The School of Global Development, University of East Anglia, Norwich NR4 7TJ, United Kingdom

**Keywords:** uncertainty, modeling, hydrology, sustainability, agriculture

## Abstract

A prevailing notion in sustainability science is that irrigated agriculture underpins global food and water security because it accounts for 40% of crop production and 70% of freshwater withdrawals. Through a network citation analysis of 3,500 documents, we reveal that this belief has spread through the literature with minimal empirical support: 60–80% of all citation paths lead to sources that lack supporting data or that do not even contain the 40 or 70% numbers. We also demonstrate that these figures mask a much more uncertain contribution of irrigation to global crop production and water withdrawals, which can lie anywhere between 18–50 and 45–90%, respectively. These ranges should be understood as lower bounds on the true uncertainty. Our findings underscore the need to rigorously evaluate foundational claims in sustainability science and embrace ambiguity to produce robust research and policy-making.

Significance Statement The assumption that irrigation agriculture withdraws 70% of all freshwater resources and produces 40% of all crops worldwide guides science and policy making on water and food security. However, the robustness of this belief is unknown. Here, we show that the 70 and 40% numbers have spread through amplification and poor citation practices, and that only ∼1.5% of the documents cited supporting these numbers provide actual data. We also reveal that the contribution of irrigation to water withdrawals and food production is much more uncertain and ranges between 45–90 and 18–50%, respectively. Our results highlight the need to revisit key tenets of sustainability science to ensure more robust insights and effective policies for water and food security

“Irrigation agriculture produces 40% of all crops and withdraws 70% of all freshwater resources worldwide.” This statement has been reiterated over the last 50 years in more than 3,000 scientific and policy-making documents and is foundational in defining belief in irrigation as key for global food and water security (1–4). Both numbers feed into research and governance: for example, the 40% figure is cited to suggest that limiting irrigation could significantly impact food security ((5), p. 1) or that we need to invest in research to achieve “more crop per drop” ((6), p. 39). As for the 70% figure, it is used by models like CLM4.5 as a numeric input to calculate the target soil moisture content ((7), pp. 358–359) and by the World Bank to argue that, given its large share, we will have to reallocate water from irrigation to the industrial and domestic sectors to meet future water demand (8). However, despite the importance of the 40 and 70% figures, we do not know how or when they were produced, how they spread and how robust they are.

Here, we address these knowledge gaps through network citation analysis (9, 10) and uncertainty and sensitivity analysis (11, 12). We compile 3,693 unique documents from 1966 to 2024 containing or cited for containing the claim that “irrigation produces 40% of all crops” (food belief network, 768 documents) and/or “irrigation withdraws 70% of all freshwater resources” (water belief network, 2,925 documents). To check the genesis and spread of these beliefs, we treat each document stating the 40% or the 70% figure as a node, each citation made to support the statement as an edge, and the citation pattern as a directed graph (9, 10). Most documents are journal articles, book chapters or scientific publications presenting original research, followed by policy documents from the Food and Agricultural Organization of the United Nations (FAO), World Bank or the United Nations, and reviews (Fig. S1, Methods). When not resulting from a specific targeted research, the 40 and 70% figures are generally mentioned in the first lines of the Introduction section to highlight the scientific and societal relevance of the study that follows, and are rarely disputed. The very few criticisms that we have documented focus on their sweeping nature ((13), p. 1) or shallow conceptualization ((14), p. 186), leaving their empirical support largely uncontested.

Our results indicate that the 40 and 70% figures propagated through the literature by hearsay and unwarranted amplification. We find that only ∼1.5% of the documents cited to support these numbers provide original data and that 60–80% of all citation paths lead to documents without data or to dead-ends; that is, to documents that do not even contain the numbers. Furthermore, we reveal that the 40 and 70% figures hide large uncertainties as irrigation’s share in global food production and freshwater withdrawals can be anywhere from 18–50 and 45–90%, respectively, given available data. These findings demonstrate that a central tenet in sustainability science has disseminated through poor citation practices rather than through rigorous testing, and that its empirical base permits a broader scope of insights and policies than currently considered. We anticipate our work to be a starting point for a more robust production of knowledge on irrigation agriculture and its global role in balancing resource use and food security.

## Results

### Genesis and authority

**Fig. 1.**
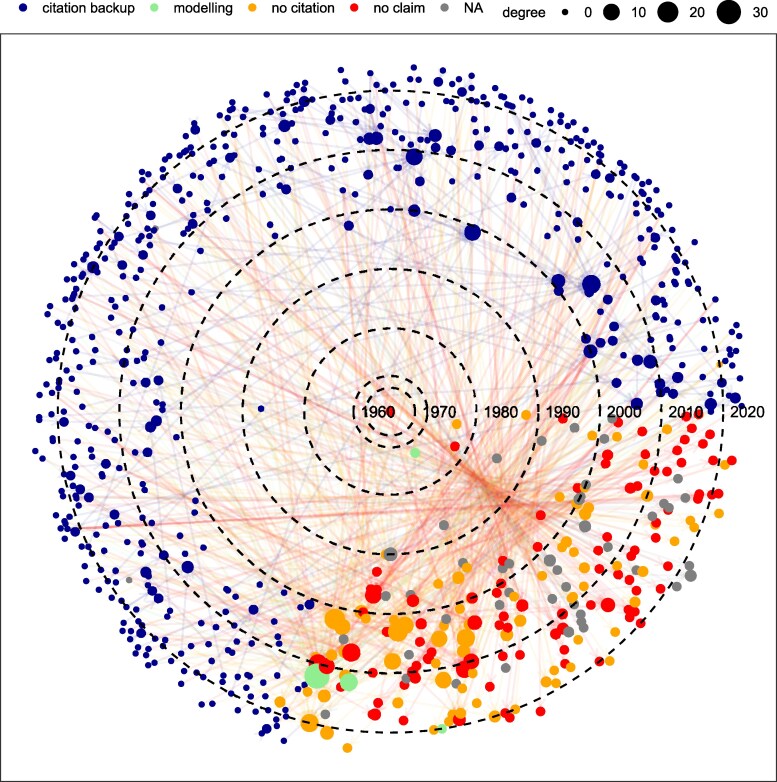
Food belief network as a function of time in polar coordinates. The size of the nodes (documents) correspond to their degree; that is, the number of incoming edges (citations). Blue nodes (“citation backup”) denote documents that make the claim and support the claim with a citation. Green nodes (“modeling”) represent documents that produce original data supporting the claim through a modeling or statistical exercise. Orange nodes (“no citation”) are documents that make the claim but do not produce original data nor cite any study to support the claim. Red nodes (“no claim”) are documents that are cited to support the claim but do not actually make the claim. Grey nodes (“NA”) represent documents that are cited as making the claim but that we have been unable to access.

**Fig. 2.**
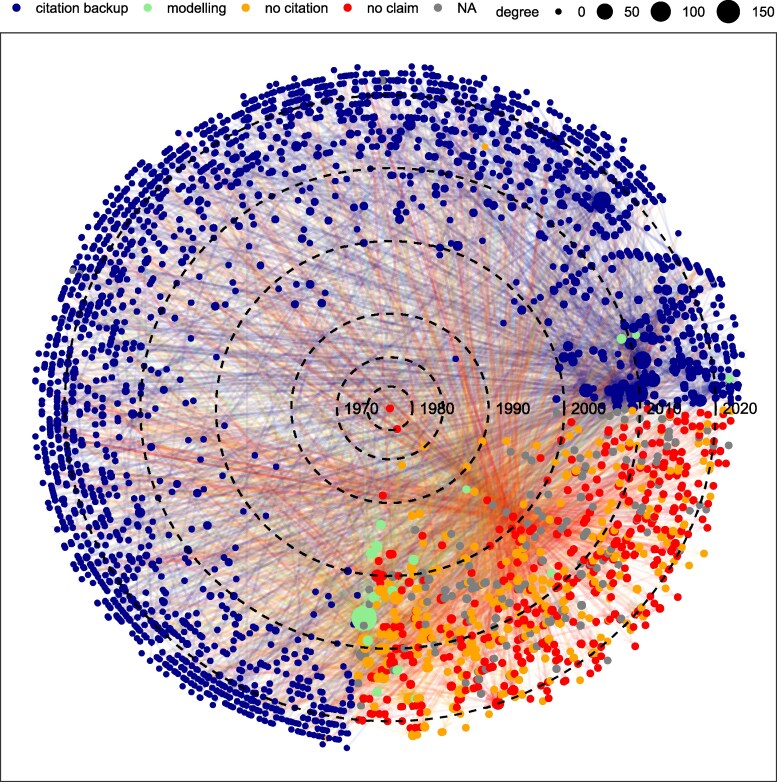
Water belief network as a function of time in polar coordinates. See caption of Fig. 1 for a description of the legend.

The food and water belief systems form two sparse networks which only share 4% of the documents (Figs. S2–S4). Their genesis is hard to trace: in each network, ∼30% of nodes represent the final link in the chain of citations for the claim (the endpoint), giving the impression that the 40 and 70% figures have received widespread independent validation. However, only ∼1.5% of this 30% provides supporting data, indicating that the claims have been widely misattributed and that the beliefs are far less corroborated by direct evidence than they appear. Most of the oldest texts (1966–1985) cited as making the claim either lack the claim or contain the claim without citing the source nor producing primary data to support it (Figs. 1–2, S5, and S6).

The earliest attested primary source for the food belief network is L’vovich ((15), pp. 313–314), who addressed grain rather than total agricultural production. He estimated that 40% of the grain supply in the year 2000 would need to come from irrigation based on assumptions about land use, yields, and the balance between grain and nongrain crops (Supplementary materials). As for the water belief network, the oldest primary documented study is The World Resources Institute (1992) (16), who did not draw on global data but on country-level statistics from the National Geological Survey (France), the US Geological Survey (USA), and the National Academy of Science (USSR). We have documented other works between 1974 and 1990 that roughly approximated the 70% figure based on water use calculations per sector, but they are not cited in the network (17, 18). In any case, Refs. (15) and (16) are cited only once and therefore probably played a minor role in shaping and spreading the food and water beliefs.

The most cited document and hence the top authority for both belief systems is FAO’s Aquastat (1). It is cited for the first time in the food and water beliefs network in 2012 and 2003, respectively, and attracts ∼5% of all direct citations. Created in 1994 as a branch of the Global Water Information System (GWIS) by FAO’s Land and Water Division, Aquastat has been collecting and producing country-level statistics on water resources and agriculture since and thus it is a primary source for both claims. Overall, Aquastat jointly with the next top nine authorities conform <1.5 and 0.3% of all nodes in the food and water network, respectively, but attract ∼20% of all direct citations, acting as endpoints for ∼25% of all citation paths (866 and 5,329, respectively) (citation paths are sequences of citations that connect documents in the network; e.g. there are three citation paths in the citation chain A→B→C; A→B, B→C, and A→B→C). However, none of the first 10 authorities besides Aquastat or Faostat (19) provides primary data to support the 40 or 70% claim: they either cite other studies (20, 21), make the claim but do not cite any source (3, 22) or do not actually make the claim (2, 23) (Table S1). The food and water beliefs have therefore been significantly pushed forward by studies that do not contain data on claim validity, a phenomenon known as “amplification” (9, 10).

### Amplification and transmutation

**Fig. 3.**
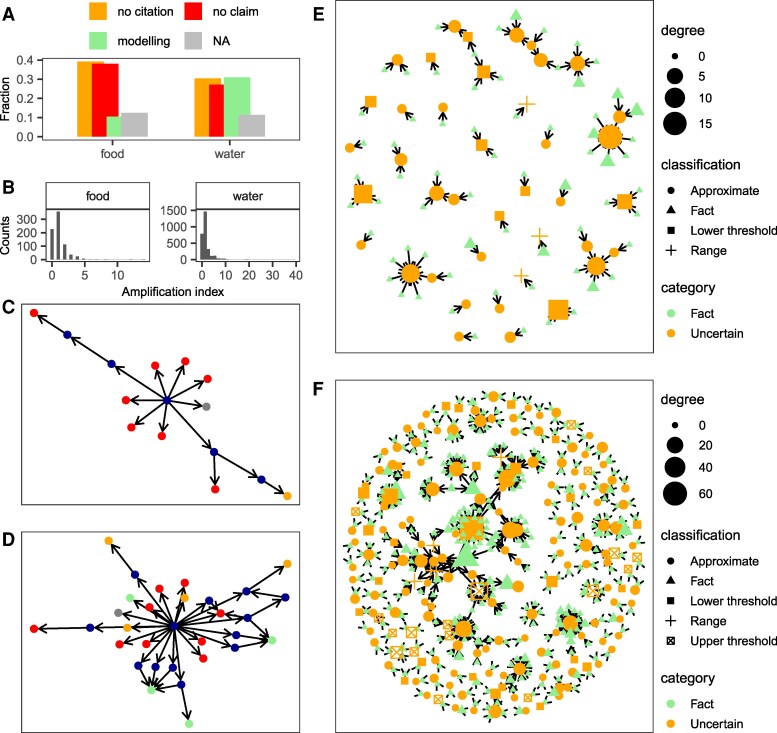
Amplification in the food and water networks. A) Fraction of all citation paths ending in primary data documents (“modeling”), dead-ends (“no claim”), documents that make the claim but do not cite nor provide data (“no citation”) or documents that we have been unable to access (“NA”). B) Distribution of the amplification index (see Methods for its calculation). C) Citation paths initiated by the paper with the highest amplification index in the food network. D) Citation paths initiated by the paper with the highest amplification index in the water network. E) Uncertain claims transformed into facts through citation alone in the food network. The degree legend shows the number of incoming citations for each document. F) Same, but for the water network.

To examine the extent to which the food and water belief systems have expanded due to amplification, we compute the proportion of citation paths that do not end in a document presenting or producing data supporting the claim. We find that 40 and 30% of all citation paths in the food and water belief networks are dead-ends; that is, finalize in documents that do not contain the claim ((10), p. 8) (Fig. 3A). An example is the United Nations’ report “World Water Development Report 2014: Water and Energy,” directly cited at least six times for the 40% claim without the report containing the figure. Since we also find that 40 and 30% of all citation paths end in documents making the claim but without citations nor primary data, ∼80 and 60% of the food and water belief systems seem to have formed through amplification. These are conservative figures given that 11% of all citation paths end up in documents which we have been unable to access and hence they can either be sources of primary data, papers without data nor citations to support the claim, or dead-ends.

We identify which documents have contributed to amplify the beliefs the most by calculating how many simple paths of length greater than 1 originate in node *v* and do not end in a primary data node (“modeling” node) (Methods). We find that the average document in the food and water networks respectively initiates 1.3 and 1.8 citation paths that do not lead to primary data documents (for a minimally amplified network this measure would equal zero ((9), p. 15 SM)). However, the distribution of the amplification index is right skewed and hence there are a few documents that significantly contributed to amplify the claims (Fig. 3B). The document that amplifies the food belief the most initiates 14 citation-paths without any ending in a “modeling” paper (24), and six out of its seven direct citations are to dead-end documents (Fig. 3C). In the case of the water belief, one document supports the claim with 23 citations (eight leading to dead-ends and two to documents with no citation), initiating 39 citation paths that do not culminate in any primary document (25) (Fig. 3D). Overall, amplification is primarily driven by journal articles, books, and other scientific publications, as policy documents rarely cite sources for the claim.

We also note a phenomenon known as “transmutation,” the transformation of a claim initially made as a nuanced statement into a sharp fact through citation alone without the production of new data ((9 ), p. 19 SM). This shift involves turning claims such as “approximately 70%” into “70%” (e.g. (26), p. 107419 citing (27)), or converting uncertain ranges, whether explicit (“30–40%”) or implicit (“up to 40%”), into precise figures. This is illustrated by document ((28), p. 62), which claims that “irrigated agriculture accounts for 40% of global food production” and cites a document where the 40% figure is actually a lower bound (“irrigated agriculture produces 40–45% of the world’s food”) (29). Similar examples can be found for the 70% number (e.g. (30), p. 278 citing (31), p. 1864). Factual claims have indistinctly been derived from claims where the 40% or the 70% values were initially presented as lower limits (e.g. (32) citing (33)), upper limits ((34) citing (22)) or were embedded in a range ((35) citing (36 )). We also note the conversion of upper into lower thresholds (e.g. (37) citing (22)) and that the 70% value is reported as both a lower and an upper bound in the water belief system (e.g. (38, 39)), which is ontologically inconsistent. Overall, the transformation of claims nuancing or acknowledging uncertainty into factual claims characterizes 9% of all citation paths in both the food and the water belief networks (Fig. 3D and E). Since ∼8% of all citation paths involves the reverse phenomenon (factual claims turned into uncertain by citation alone), roughly one in seven citation paths in the networks alters the strength of the cited claim.

A more subtle distortion happens when a citation alters the meaning of the claim. For instance, one study argued that irrigated croplands provide ∼40% of global food production (40), but the study it cited refers to the 40% as the percentage of total calories produced by irrigation (41). By shifting the focus from calories to food production (a broader term), the document suggests that irrigation supports a wider range of agricultural output (including livestock) than implied in the cited source. In the water belief network, a common distortion involves turning the 70% figure from a number describing global water withdrawals (which include a fraction of water that is recoverable) to global water consumption (which denotes the water depleted from, and unavailable to, the system), or vice versa (e.g. (42) citing (43)). This conflation leads to a misunderstanding of the connection between irrigation and catchment water scarcity: while 70% withdrawals by irrigation may not impact other sectors provided much of that water returns to water systems, 70% consumption is a considerable volume suggesting many catchments are closed and have little spare water to share. The confounding between withdrawals and consumption has been noted elsewhere (44).

### Empirical support

The 40 and 70% numbers largely spread through amplification, transmutation and poor citation practices, but how solid are the data? To address this question, we focus on the most cited authority for both beliefs, Aquastat (1). We retrieve its data on the percentage of total irrigated grain production and on the percentage of agricultural water withdrawals between ∼1990 and 2020.

**Fig. 4.**
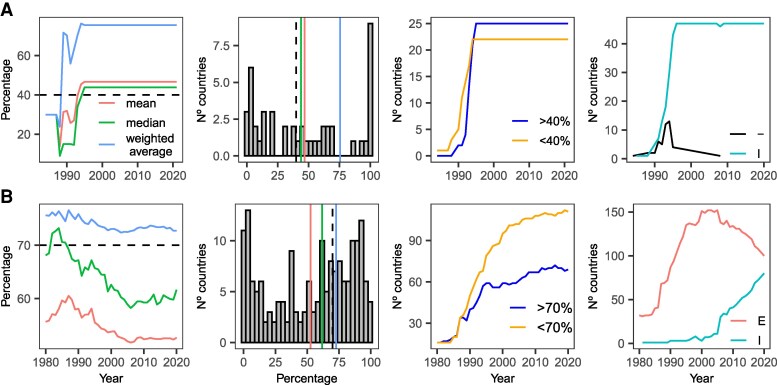
Aquastat data (1). A) Data on the percentage of total grain production that is irrigated, corresponding to the food belief system (40%). B) Data on the percentage of agricultural water withdrawals, corresponding to the water belief system (70%). In the first column, the global mean, median, and weighted average are taken from data at the country level. Plots in the second column display the distribution of values at the country level for 2020. The vertical dashed line is at 40 and 70%, respectively. Plots in the third column display the number of countries whose proportion of total irrigated grain production (agricultural water withdrawals) is above or below 40% (70%). Plots in the fourth column show the number of countries for which the data is imputed (I), estimated (E) or for which there is no information on the calculation (−). According to Aquastat, estimated values are calculated as sum or identified from official values or from an Aquastat estimation. Imputed values are those values which have been carried over from previous years, linearly interpolated between two existing official data-points or imputed based on their latest respective share.

For the food belief, the 40% figure approximates the average or the median percentage of irrigated grain in the world (Fig. 4A). However, Aquastat’s dataset consists of only one country in 1984 (Somalia), seven countries in 1990 and 47 countries from 1995 onward (25% of the total number of countries, c. 190), with no countries from Europe or the Americas and half of the countries in Asia and Africa. Almost all data are imputed (not measured nor simulated) and values have been carried over since 1995 without any update. For the water belief, a weighted average is the summary statistic that better approximates the 70% claim, as both the median and the mean are far off (Fig. 4B). This indicates that Aquastat’s data only supports an interpretation of the 70% figure as a number reflecting global trends with a clear focus on countries that are major water users, not as a global summary statistic.

Aquastat might have been widely cited as the primary authority on the food and water beliefs more for its scientific and societal influence on agriculture-related topics than for its data genuinely supporting these beliefs. It is thus possible that solid support for the 40 and 70% figures exists, but that it spread informally rather than through academic citations. To investigate this possibility, we collect all data available that might have played a part in the production of the claims and reverse-engineer the calculations behind the 40 and 70% figures in a global uncertainty and sensitivity analysis (UA/SA). Overall, we consider data from eleven global hydrological models, two FAO-based datasets and 13 independent datasets on irrigated and nonirrigated areas and crop production (Methods, Supplementary materials). We assume that a commitment to the 40 and 70% figures and hence to the food and water beliefs is warranted if the numbers hold once uncertainties across datasets, models and methodologies are propagated in the calculation.

**Fig. 5.**
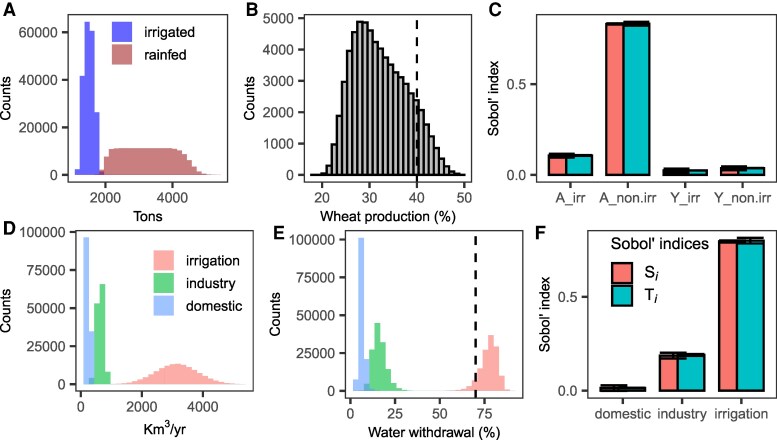
Uncertainty and sensitivity analysis. A) Uncertainty in the global average production of wheat in irrigated and nonirrigated land. See Figs. S10 and S11 for maize. B) Uncertainty in the percentage of total wheat produced by irrigated agriculture. C) Sobol’ indices. First-order effects ($S_{i}$) describe the fraction of uncertainty conveyed to the estimation by each uncertain parameter, whereas total-order effects describe first-order effects and the effects derived from interactions with the other parameters. The error bars show the 95% CI, computed with the percentile method. When $S_{i}=T_{i}$, the parameter is not involved in interactions. D) Distribution of the volume of water withdrawn by irrigation, industry, and the domestic sector. E) Percentage of water withdrawn by each sector. The vertical, dashed line is at 70%. F) Sobol’ indices.

With current data, the percentage of wheat and maize (two leading grains in terms of output) produced under irrigation agriculture ranges between 18 and 50% ($Q_{2.5,97.5}=[23,44]$%), placing the 40% figure near the upper bound of the uncertainty distribution (Fig. 5A and B). Only 30% of our simulations fall within the 40±5% range and 80% yield percentages below 40%. The primary source of uncertainty in estimating the percentage of irrigated grain production is the extent of nonirrigated land (A_non_irr) (Fig. 5C), which ranges between 700 Mha and 1,600 Mha (45, 46) and contributes close to 80% of the variance. The extension of nonirrigated (rainfed) land is needed to calculate the total extension of agricultural land, from which the proportion of irrigated grain can then be derived. The second is the area under irrigation (A_irr), ranging from 295 Mha to 400 Mha (21, 47–49) and contributing about 10% of the variance. The uncertainty in the average global production of irrigated (wheat=∼4–5 tons/hectare, maize = ∼6–9 tons/hectare; Y_irr) and nonirrigated (wheat=∼3–4 tons/hectare, maize = ∼3–6 tons/hectare; Y_non_irr) grain has a negligible effect (Figs. 5A–C and S7). These results hold when shifting the focus from the percentage of irrigated grain production to the percentage of calories produced from irrigated grain (Figs. S8 and S9).

As for the percentage of irrigation water withdrawals, the distribution is strongly left skewed and ranges from 45 to 90% ($Q_{2.5,97.5}=[70,85]$%) (Fig. 5d–e), with 25% of the simulations falling within the 70±5% range. Estimates of irrigated agriculture water withdrawals range between 1,000 and 5,000 km${\;}^{3}$/year of water and consistently dominate global withdrawals, though sectoral shares vary: when irrigation accounts for ∼90% of freshwater withdrawals, industry and domestic sectors account for 5–6 and 3–4%. When irrigation withdrawals drop to ∼50%, industry and domestic withdrawals rise to ∼35% and ∼15%. Almost all the uncertainty in determining irrigation’s withdrawals as a percentage of total water withdrawals stems from the ambiguity in estimating the volume withdrawn by irrigation, followed by that of industry (Fig. 5D–F). The uncertainty in domestic withdrawals does not have an effect. Overall, total water withdrawal for domestic, industrial and irrigation purposes ranges from 1,700 to 6,500 km${\;}^{3}$/year, reflecting an uncertainty spanning half an order of magnitude.

## Discussion

The belief that irrigation produces 40% of all food and withdraws 70% of global freshwater has limited direct evidence and has gained traction through misattributed claims, amplification, and poor citation practices. Current data indicate a more nuanced role for irrigation, with estimates for the percentage of total grain production ranging from 18 to 50% ($Q_{2.5,97.5}=[25,45]$%) and freshwater withdrawals from 45 to 90% ($Q_{2.5,97.5}=[70,85]$%). These ranges likely underestimate the true uncertainty because they derive from available estimates whose production has not undergone an uncertainty and sensitivity analysis (UA/SA). For instance, the water withdrawal data that we have used in our uncertainty analysis is produced by models that only use one map of irrigated areas (the FAO-GMIA (21)), even though at least four other maps exist which provide values for the same irrigated grid cell that can vary by up to two orders of magnitude (50, 51). Those models also associate specific irrigation technologies (drip, sprinkler, flood) with fixed efficiency values, resulting in overly precise withdrawal estimates at the country-level that miss uncertainties by an order of magnitude (52). Had these ambiguities been incorporated into the models, their estimations would have had a much larger variability and this variability would have propagated in our uncertainty analysis, leading to larger ranges due to the “uncertainty cascade” effect (53).

The 40 and 70% figures are an instance of sustainability- and policy-relevant numbers that have circulated widely without scrutiny, but there are others. For example, in 2017, the claim that 100 companies were responsible for 71% of global emissions spread through outlets like The Guardian and CNBC (54, 55), even though the original study focused solely on fossil fuel and cement production (thus excluding emissions from land use, agriculture, or indirect emissions) (56). In 2018, the belief that Americans used 500 million plastic straws daily spurred bans by Starbucks and Marriott, only to trace back to a 9-year-old’s estimate based on the assumption that each person consumed 1.5 plastic straws (57). The propagation of the 40 and 70% numbers took place over the last 50 years and accelerated post-2000 (Fig. S12), and currently few irrigation studies omit them. Overall, the passive embrace of the 40 and 70% figures over five decades aligns with the lack of critical research in water studies noted by *Nature Sustainability* in 2021 (58).

Numbers with relevance for sustainability spread profusely because they elicit a strong emotional response, provide authors access to an epistemic community and permit a seemingly straightforward understanding of complex challenges. The 40:70 duplet conveys a clear picture of the relevance of irrigation for global food and water security and beguilingly simplifies the policy action landscape. Yet our findings reveal a more nuanced reality, encapsulated within four policy scenarios or quadrants that emerge from combining the extreme values within the uncertainty ranges of the food and water beliefs:

- Quadrant 1: Low food production (∼18%) and low water withdrawals (∼45%): Irrigation has a minimal role in global food security. Improving water efficiency to reduce water consumption has limited impact and hence may not be a top priority.
- Quadrant 2: Low food production (∼18%) and high water withdrawals (∼90%): Irrigation may be inefficient. There is an urgent need to boost economic and productive efficiency to prevent water scarcity with low food returns.
- Quadrant 3: High food production (∼50%) and low water withdrawals (∼45%): Irrigation is very effective for global food and water security, maintaining productivity and efficiency is essential; a watchful “business as usual” scenario is suggested.
- Quadrant 4: High food production (∼50%) and high water withdrawals (∼90%): Irrigation supports high food production at high water costs. Hence, we need to prioritize sustainable water management practices to sustain food production but cut water consumption.

The range of possibilities within these scenarios highlights the complexity of the science-policy interface when fundamental uncertainties are explicitly accounted for. All four quadrants and the scenarios “in the middle” are supported by available data. Under these circumstances, definitive science-based decisions are often elusive and sustainability research is more valuable for fostering skepticism and expanding evidence than for closing down debate (59). Embracing uncertainty helps keeping the range of possible futures open by challenging narratives that present themselves as the only way forward given the science available. One example is the claim that global market-based water policies are urgently needed to bring humanity back to a freshwater “safe space,” a policy recommendation based on a model that collapsed uncertainties in diets and undernourishment into the assumption that everyone on Earth consumes 3,000 kcal daily ((60), p. 384).

Our results suggest that initiatives addressing water challenges should recognize the difficulties of quantifying irrigation’s role in global food and water security and focus on developing policies that remain solid regardless of an accurate determination of food-water quanta. Sharper estimates of irrigation’s role in global crop production and water withdrawal are unlikely: firstly, the ranges unfolded here are likely to expand when uncertainties in the calculation are fully accounted for, as already discussed. And secondly, attempts to reduce uncertainties with more detailed models or irrigation datasets often have the opposite effect (53), as 50 years of research on global irrigation water withdrawals show (61). Efforts to pin down irrigation’s role in global food and water security are less about finding “real numbers” than about our reliance on quantification *über alles* as the basis for policy. Governance by numbers makes the complexity of irrigation legible by policy-makers while encoding under seemingly objective figures normative assumptions, power relations, institutional preferences, and epistemological commitments (e.g. what needs to be measured and how) (62).

Acknowledging the need to research the links between irrigation, water and food production, one possible way forward is to prioritize smaller-scale and place-based initiatives over global solutions based on numbers (63), which falter when the starting point is unclear. This approach, ideally using multiple methods, can engage with local land-water resources, stakeholders, and democratic deliberation, potentially leading to more robust outcomes. For example, in Pickering (United Kingdom), stronger authority–stakeholder relationships emerged when stakeholders defined action by co-designing a water management model that relegated “scientific validation” as secondary (64). In Tanzania, methods were proposed for irrigators to gain insights for improving crop production and water use by simply comparing their practices with their neighbors’, relying on peer-driven learning rather than on benchmark-based calculations (65). Clearly, reducing water consumption and boosting crop production are desirable goals because of climate change, growing competition for water and increasing food needs. If the figures defining the role of irrigation in global food and water security have spread unwarrantedly and are ambiguous, achieving these goals may require abandoning global numbers and the global scale altogether.

## Methods

### Creation of the corpus

We examined two central claims in food and water security: that irrigation agriculture produces 40% of all crops consumed worldwide and that irrigation agriculture withdraws 70% of all freshwater resources. Based on Refs. (9, 10), we treated these claims as beliefs formed through repeated citations and studied their emergence and dissemination via network citation analysis.

We used the Dimensions database to identify studies potentially making either of these two claims. Unlike Scopus and the Web of Science, Dimensions allows search queries not only in the title, abstract, and keywords but also in the full text when it is indexed in the background. Initially, we tested various search queries with different keywords and logical operators to check the ratio of false positives per search query (i.e. studies returned by the search but lacking the claims in the main text). From this preliminary analysis, we identified the most common keywords accompanying the claims, analyzed their distances within the claim and refined the search queries until we converged towards the following two proximity searches:

1. (“agriculture water 70 global”∼n) OR (“irrigation water global 70”∼n) OR (“agriculture water 70 world”∼n) OR (“irrigation water 70 world”∼n)
2. (“agriculture food 40 global”∼n) OR (“irrigation food global 40”∼n) OR (“agriculture food 40 world”∼n) OR (“irrigation food 40 world”∼n),

where *n* is the maximum number of words separating the keywords, which we set at n=25. These search queries returned 3,090 and 3,734 references for the food and water belief system, respectively, which we denoted as our “work” corpus. For each reference in the corpus, we retrieved the main text in either PDF or physical form and searched for explicit mentions of “70%,” “70 percent”, “40%,” or “40 percent,” either programatically or through close-reading. If no claim was found, we searched for “irrigat” to ensure claims like “irrigation withdraws between 50 and 90% of all freshwater resources” were not missed. A reference in the “work” corpus without the claim was considered a false positive and we proceeded to the next. In total, 20% (616) of the references in the food corpus and 70% (2,548) in the water corpus contained the targeted claim.

### Data collection

For those references that contained the claim, we recorded its doi/ISBN, the title of the study and the authorship. Based on Ref. (9), we also collected the following data (Fig. S13).

1. The sentence/s containing the claim. We also retrieved the preceding and following sentences if they were relevant to contextualize the sentence making the claim.
2. The type of document making the claim. We classified as “review” studies explicitly labeled as such in the journal or with “review” in their title; and as “policy” documents published by policy-oriented institutions like the FAO, World Bank or the International Water Management Institute (IWMI). Finally, we denoted with “others” all remaining types of documents, including journal papers, book chapters, books, conference proceedings, or PhD dissertations.
3. The nature of the claim. If a study produced primary data to support the claim (either through a modeling or statistical exercise), we labeled the study as “modeling.” If a document made the claim but cited another study to support it, we classified it as “citation backup.” If a document made the claim without supporting it with any citation, we denoted it as “no citation.” If document $v_{1}$ made the claim, cited paper $v_{2}$ to support the claim but paper $v_{2}$ did not actually contain the claim, we classified paper $v_{2}$ as “no claim.”
4. The citation supporting the claim. For each document classified as “citation backup,” we retrieved the study or studies cited as supporting the claim. We considered that a claim was cited when the citation was made directly in the sentence containing the claim or in the sentence immediately after, separated from the actual claim by a comma, not a full stop. The cited study or studies were included in the “work” corpus and treated as new entries for which data regarding the sentence containing the claim, the type of document making the claim, the nature of the claim, etc., were also collected.
5. The strength of the claim. We classified the claim as a “fact” when it was uttered assertively (“irrigation withdraws 70% of all freshwater resources”), as “approximate” when it accounted for uncertainty (“irrigation withdraws approximately 70%”, “irrigation withdraws about 70%,” “irrigation withdraws c. 70%”), as “upper” if the number in the claim reflected an upper bound (“irrigation withdraws up to 70% freshwater resources”), as “lower” if the number in the claim denoted a lower bound (“irrigation withdraws above 70%”), and as “range” if the numbers in the claim encompassed the number of interest without mentioning it (“irrigation withdraws between 50 and 80% of all freshwater resources”).

### Network analysis

The final citation network for the food and water belief systems consisted of 764 nodes (documents) and 660 edges (citations) and 2,926 nodes and 2,803 edges, respectively, spanning the years 1966–2024 for food and 1977–2025 for water. We represented each network or graph as G(V,E), where *V* is the set of nodes, and *E* is the set of directed edges between nodes, and analyzed the network with the R packages igraph (66) and ggraph (67). To determine the number of citations that each paper cited as making the claim received, we computed the in-degree metric for each node *v*, defined as the number of incoming edges, or


(1) in-degree(v)=|{u∈V∣(u,v)∈E}|,

where (u,v)∈E represents a directed edge from node *u* to node *v*.

To calculate the proportion of paths ending in nodes whose nature of claim was either “no citation” or “no claim,” we first computed the number of edges (i.e. citations) going out of each node, the out-degree attribute, and defined a terminal node as a node whose out-degree(v)=0. We then identified the set of terminal nodes that did not make the claim as


(2) $$V_{\mathrm{no}\_\mathrm{claim}}=\{v\in V|\text{out-degree}(v)=0\;\text{and}\;\text{nature claim}(v)=\text{''no claim''}\},$$

and the set of terminal nodes that made the claim but did not include a citation as


(3) $$V_{\mathrm{no}\_\mathrm{citation}}=\{v\in V|\text{out-degree}(v)=0\;\text{and}\;\text{nature claim}(v)=\text{''no citation''}\}.$$

For each terminal node in either $V_{\mathrm{no}\_\mathrm{claim}}$ or $V_{\mathrm{no}\_\mathrm{citation}}$, we defined all its predecessor nodes (any node that has a directed path leading to the terminal node) as


(4) Predecessor(v)={u∈V∣u→vin a directed path},

where Predecessor is the set of predecessor nodes for terminal node *v* and *u* is any node that is upstream (in terms of directed edges) from *v*, meaning that there is a sequence of directed edges in the graph *G* that starts from *u* and ends at *v*. The set of all unique predecessors for a set of terminal nodes *T* is then defined as


(5) $${\text{Predecessor}}_{\mathrm{unique}}(T)=\underset{v\in T}{⋃}\text{Predecessor}(v),$$

where T⊆V is the set of terminal nodes, i.e. nodes with no outgoing edges, Predecessor(v)={u∈V∣u→v} is the set of predecessor nodes of terminal node *v*, and $P_{\mathrm{unique}}(T)$ represents the unique set of all predecessor nodes across all terminal nodes in *T*. Finally, we calculated the proportion of paths leading to nodes classified as “no claim” or “no citation” as


(6) $$\begin{array}{cc} P_{\mathrm{no}\_\mathrm{claim}} & \;\;\;\;\;\;=\frac{\left|{\text{Predecessors}}_{\mathrm{no}\_\mathrm{claim}}\right|}{\left|V\right|}\\ P_{\mathrm{no}\_\mathrm{citation}} & =\frac{\left|{\text{Predecessors}}_{\mathrm{no}\_\mathrm{citation}}\right|}{\left|V\right|}, \end{array}$$

where ∣V∣ is the cardinality of set *V*.

To calculate the amplification index for each v∈V, we defined the set of nodes which support the claim with a modeling exercise *M* as


(7) M={v∈V∣nature claim(v)=''modeling''}

Let P(v) denote the set of all simple paths that originate in *v*. Since we exclude paths *ρ* of length 1 that lead to a modeling node m∈M, hence


(8) $$P_{\mathrm{filtered}}(v)=\{\rho \in P(v)|\text{length}(\rho )>1\;\text{or}\;\text{end}(\rho )\notin M\},$$

where $P_{\mathrm{filtered}}$ is the set of paths of length greater than 1 that do not end in a modeling node, length(ρ) is the number of edges in the path *ρ* and end(ρ) is the terminal node of the path *ρ*. The final amplification index (AI) is then calculated as


(9) $$\text{AI}(v)=|P_{\mathrm{filtered}}(v)|,$$

where $P_{\mathrm{filtered}}(v)$ denotes the cardinality of the set $P_{\mathrm{filtered}}(v)$.

### Uncertainty and sensitivity analysis

To assess whether the available data supports the belief that irrigation produces 40% of all crops and withdraws 70% of all freshwater resources, we conducted a Monte Carlo-based uncertainty and sensitivity analysis. For each belief, we defined a *d*-dimensional hypercube with $N_{s}$ sampling points produced with Sobol’ quasirandom numbers (68) and represented by the matrix X, such that


(10) $$X=\left[\begin{array}{cccc} x_{1}^{\left(1\right)} & x_{2}^{\left(1\right)} & \cdots & x_{d}^{\left(1\right)}\\ x_{1}^{\left(2\right)} & x_{2}^{\left(2\right)} & \cdots & x_{d}^{\left(2\right)}\\ \vdots & \vdots & \ddots & \vdots \\ x_{1}^{\left(N_{s}\right)} & x_{2}^{\left(N_{s}\right)} & \cdots & x_{d}^{\left(N_{s}\right)} \end{array}\right],$$

where $x_{j}^{\left(i\right)}$ is the value taken by the *j*th column in the *i*th row and column $x_{j}$ reflects the uncertainty in parameter $X_{j}$. For the food belief, the X matrix had four columns corresponding to the four uncertain parameters involved in the calculation of the percentage of grain produced under irrigation agriculture, which we estimate in the *i*th simulation as


(11) $$p_{\mathrm{grain},\mathrm{irr}}^{\left(i\right)}=100\left(\frac{y_{\mathrm{irr}}^{\left(i\right)}a_{\mathrm{irr}}^{\left(i\right)}}{y_{\mathrm{irr}}^{\left(i\right)}a_{\mathrm{irr}}^{\left(i\right)}+y_{\mathrm{nonirr}}^{\left(i\right)}a_{\mathrm{nonirr}}^{\left(i\right)}}\right),$$

where $p_{\mathrm{grain},\mathrm{irr}}$ represents the grain produced in irrigated areas [%], $y_{\mathrm{irr}}$ and $y_{\mathrm{noirr}}$ are the yields per unit area in irrigated and nonirrigated land [Tons/ha], and $a_{\mathrm{irr}}$ and $a_{\mathrm{nonirr}}$ denote the total areas of irrigated and nonirrigated land [Mha]. We described each of these four parameters with probability distributions based on the uncertainty defined after collecting data from 13 different datasets (Tables S2 and S3). The result is a vector of values denoting the uncertainty in the percentage of grain produced under irrigation across all simulations, defined as


(12) $$p_{\mathrm{grain},\mathrm{irr}}=\left[\begin{array}{c} \;p_{\mathrm{grain},\mathrm{irr}}^{\left(1\right)}\\ p_{\mathrm{grain},\mathrm{irr}}^{\left(2\right)}\\ \vdots \\ p_{\mathrm{grain},\mathrm{irr}}^{\left(N_{s}\right)} \end{array}\right].$$

With regards to the water belief, we defined three sectoral X matrices (corresponding to industrial, domestic and irrigation withdrawals), each with 140 columns as we retrieved data for 140 countries. Based on the data collected from seven independent datasets and 12 global models (Supplementary materials), we describe the uncertainty in column *j* as $U({\text{min}}_{j},{\text{max}}_{j})$, where min${\;}_{j}$, max${\;}_{j}$ denote the minimum and maximum industrial, domestic, or irrigation water withdrawal values for country *j*.

For $i=1,2,\ldots ,N_{s}\in X$, we computed the sum of all entries across the *d* columns to obtain the corresponding total water withdrawals, as


(13) $$y_{\mathrm{sector}}^{\left(i\right)}=\sum_{\;j=1}^{d}x_{j}^{\left(i\right)},$$

where $y_{\mathrm{sector}}^{\left(i\right)}$ is the sum of the *d* values in the *i*th row of X for each specific water use sector. We then define the three vectors of row-wise sums, representing the uncertainty in global industrial, domestic, and irrigation withdrawals as follows:


(14) $$y_{\mathrm{ind}}=\left[\begin{array}{c} y_{\mathrm{ind}}^{\left(1\right)}\\ y_{\mathrm{ind}}^{\left(2\right)}\\ \vdots \\ y_{\mathrm{ind}}^{\left(N_{s}\right)} \end{array}\right],\;y_{\mathrm{dom}}=\left[\begin{array}{c} y_{\mathrm{dom}}^{\left(1\right)}\\ y_{\mathrm{dom}}^{\left(2\right)}\\ \vdots \\ y_{\mathrm{dom}}^{\left(N_{s}\right)} \end{array}\right],\;y_{\mathrm{irr}}=\left[\begin{array}{c} y_{\mathrm{irr}}^{\left(1\right)}\\ y_{\mathrm{irr}}^{\left(2\right)}\\ \vdots \\ y_{\mathrm{irr}}^{\left(N_{s}\right)} \end{array}\right].$$

We then compute total global water withdrawals as


(15) $$y_{\mathrm{total}}=\left[\begin{array}{c} y_{\mathrm{total}}^{\left(1\right)}\\ y_{\mathrm{total}}^{\left(2\right)}\\ \vdots \\ y_{\mathrm{total}}^{\left(N_{s}\right)} \end{array}\right]=\left[\begin{array}{c} y_{\mathrm{ind}}^{\left(1\right)}+y_{\mathrm{dom}}^{\left(1\right)}+y_{\mathrm{irr}}^{\left(1\right)}\\ y_{\mathrm{ind}}^{\left(2\right)}+y_{\mathrm{dom}}^{\left(2\right)}+y_{\mathrm{irr}}^{\left(2\right)}\\ \vdots \\ y_{\mathrm{ind}}^{\left(N_{s}\right)}+y_{\mathrm{dom}}^{\left(N_{s}\right)}+y_{\mathrm{irr}}^{\left(N_{s}\right)} \end{array}\right].$$

We finally produce three vector of values reflecting the uncertainty in the proportion of water withdrawn by industry, domestic, and irrigation as


(16) $$p_{\mathrm{ind}}=\left[\begin{array}{c} \frac{y_{\mathrm{ind}}^{\left(1\right)}}{y_{\mathrm{total}}^{\left(1\right)}}\\ \frac{y_{\mathrm{ind}}^{\left(2\right)}}{y_{\mathrm{total}}^{\left(2\right)}}\\ \vdots \\ \frac{y_{\mathrm{ind}}^{\left(N_{s}\right)}}{y_{\mathrm{total}}^{\left(N_{s}\right)}} \end{array}\right],\;p_{\mathrm{dom}}=\left[\begin{array}{c} \frac{y_{\mathrm{dom}}^{\left(1\right)}}{y_{\mathrm{total}}^{\left(1\right)}}\\ \frac{y_{\mathrm{dom}}^{\left(2\right)}}{y_{\mathrm{total}}^{\left(2\right)}}\\ \vdots \\ \frac{y_{\mathrm{dom}}^{\left(N_{s}\right)}}{y_{\mathrm{total}}^{\left(N_{s}\right)}} \end{array}\right],\;p_{\mathrm{irr}}=\left[\begin{array}{c} \frac{y_{\mathrm{irr}}^{\left(1\right)}}{y_{\mathrm{total}}^{\left(1\right)}}\\ \frac{y_{\mathrm{irr}}^{\left(2\right)}}{y_{\mathrm{total}}^{\left(2\right)}}\\ \vdots \\ \frac{y_{\mathrm{irr}}^{\left(N_{s}\right)}}{y_{\mathrm{total}}^{\left(N_{s}\right)}} \end{array}\right].$$

We also conducted a global sensitivity analysis using Sobol’ indices (69), which decompose the variance of the output into fractions that are attributed to the inputs, pairs of inputs, triplets etc. and up to the *d*th order. We used the Jansen estimators and implemented the sensitivity analysis using the R package sensobol (11).

## Supplementary Material

pgaf323_Supplementary_Data

## Data Availability

The code to reproduce our results, jointly with the datasets generated, are available in Zenodo (71) (https://zenodo.org/records/17038909).
