# Supplementary material for: Widely cited global irrigation statistics lack empirical support
Source: PNAS Nexus. 2025 Nov 11;4(11):pgaf323. doi: 10.1093/pnasnexus/pgaf323 (PMC12604014; doi:10.1093/pnasnexus/pgaf323)
Supplement: pgaf323_Supplementary_Data [file pgaf323_supplementary_data.pdf]

Widely cited global irrigation statistics lack empirical support

Supplementary Materials

Arnald Puy*[1], Seth N. Linga[1], Nanxin Wei[1], Samuel Flinders[1], Bethan Callow[1], Grace Allen[1], Beatrice Cross[1], Carmen Aguiló-Rivera[1], and Bruce Lankford[2]

[1] *School of Geography, Earth and Environmental Sciences, University of Birmingham, Birmingham B15 2TT, United Kingdom. E.mail: a.puy@bham.ac.uk*

[2] *The School of Global Development, University of East Anglia, Norwich NR4 7TJ, United Kingdom*

Table of Contents

1 Tables **2**

2 Calculations behind the food belief **2**

3 Uncertainty analysis **4**

4 Figures **5**

*Corresponding author

1 Tables

Table S1: The ten most cited documents for the water and the food claim (degree column).

ID	Nature of claim	Document type	Degree	Belief
fao aquastat [1]	modelling	policy	178	water
siebert et al 2010 [2]	citation backup	other	67	water
fao 2011 [3]	citation backup	policy	66	water
molden et al 2007 [4]	citation backup	policy	42	water
fao 2017b [5]	citation backup	policy	41	water
world water assessment programme 2012 [6]	citation backup	policy	32	water
foley et al 2011 [7]	citation backup	other	31	water
united nations 2003 [8]	citation backup	policy	28	water
rosegrant et al 2009 [9]	no citation	other	25	water
rost et al 2008 [10]	no citation	policy	25	water
fao aquastat [1]	modelling	policy	32	food
fao 2002 [11]	no citation	policy	18	food
morris et al 2003 [12]	no citation	policy	16	food
siebert and doll 2010 [13]	no claim	other	14	food
siebert et al 2005 [14]	citation backup	other	13	food
world bank 2020 [15]	no citation	policy	13	food
molden et al 2007 [4]	no claim	policy	13	food
abdullah 2006 [16]	no citation	other	13	food
faostat [17]	modelling	policy	12	food
doll and siebert 2002b [18]	no citation	other	11	food

2 Calculations behind the food belief

L'vovich (1974)

L'vovich [19, pp. 313–314] aimed at approximately determining the volume of agricultural products which will have to be produced to meet total human food requirements in the year 2000. He arrives at the 40% number using the numbers in Table S2 with this sequence of calculations:

Total grain yield in irrigated areas:

$$T_{g,\text{irr}} = a_{\text{irr},g} \times y_{\text{irr},g} = 150 \times 3 = 450\,. \tag{1}$$

Total non-grain yield in irrigated areas:

$$T_{ng,\text{irr}} = a_{\text{irr},ng} \times y_{\text{irr},ng} = 30 \times 3 = 90\,. \tag{2}$$

Total grain yield in non-irrigated areas:

$$T_{g,\text{non-irr}} = a_{\text{non-irr},g} \times y_{\text{non-irr},g} = 430 \times 0.8 = 344\,. \tag{3}$$

Total non-grain yield in non-irrigated areas:

$$T_{ng,\text{non-irr}} = a_{\text{non-irr},ng} \times y_{\text{non-irr},ng} = 620 \times 0.5 = 310\,. \tag{4}$$

Hence the total production in irrigated areas is

$$T_{\text{irr}} = T_{g,\text{irr}} + T_{ng,\text{irr}} = 450 + 90 = 540\,. \tag{5}$$

The total production in non-irrigated areas is

$$T_{\text{non-irr}} = T_{g,\text{non-irr}} + T_{ng,\text{non-irr}} = 344 + 310 = 654\,. \tag{6}$$

We finally arrive at the proportion of food (grain!) produced in irrigated areas:

$$P_{\text{irr}} = \frac{T_{\text{irr}}}{T_{\text{irr}} + T_{\text{non-irr}}} = \frac{540}{540 + 654} = 0.45 \approx 0.4\,. \tag{7}$$

Table S2: Variables used by L'vovich [19].

Variable	Notation	Value	Unit
Area of irrigated grain land	$a_{\text{irr},g}$	150×10^6	hectares
Area of irrigated non-grain land	$a_{\text{irr},ng}$	30×10^6	hectares
Area of non-irrigated grain land	$a_{\text{non-irr},g}$	430×10^6	hectares
Area of non-irrigated non-grain land	$a_{\text{non-irr},ng}$	620×10^6	hectares
Average yield of irrigated grain	$y_{\text{irr},g}$	3	tons/hectare
Average yield of irrigated non-grain	$y_{\text{irr},ng}$	15	tons/hectare
Average yield of non-irrigated grain	$y_{\text{non-irr},g}$	0.8	tons/hectare
Average yield of non-irrigated non-grain	$y_{\text{non-irr},ng}$	0.5	tons/hectare

FAO's Aquastat

Several FAO Aquastat regional reports offer data on irrigated grain production as a percentage of total grain production [20–23]. FAO uses the word "grain" to refer to cereals, but it does not explicit which cereals were taken into account in the calculations. Although their methodology to produce the 40% number is the same as L'vovich [19], we could not find any record of FAO citing him. FAO's chain of calculations is the following:

Total production in irrigated areas is:

$$T_{\text{irr}} = \sum_i a_{\text{irr},i} \times y_{\text{irr},i}\,, \tag{8}$$

where i indexes the crop. The total production in non-irrigated areas equals:

$$T_{\text{non-irr}} = \sum_i a_{\text{non-irr},i} \times y_{\text{non-irr},i}\,. \tag{9}$$

Hence the proportion of total irrigated production over total production:

$$P_{\text{irr}} = \frac{T_{\text{irr}}}{T_{\text{irr}} + T_{\text{non-irr}}}\,. \tag{10}$$

Rosa et al (2020)

Rosa et al. [24, p. 8] estimated that irrigation agriculture contributes 34% of total crop production by calculating the total calories produced as the product of crop yield (Tons/ha), calorie content (Kc/Tons) and harvested area (Ha). Their approach can be summarized as follows:

Total production of calories in irrigated areas:

$$C_{\text{irr}} = \sum_i c_{\text{irr},i} \times a_{\text{irr},i} \times y_{\text{irr},i}\,, \tag{11}$$

where i indexes the crop. The total production of calories in non-irrigated areas is

$$C_{\text{non-irr}} = \sum_i c_{\text{non-irr},i} \times a_{\text{non-irr},i} \times y_{\text{non-irr},i}\,, \tag{12}$$

and hence the proportion of total calories produced under irrigation is

$$P_{\text{irr}} = \frac{C_{\text{irr}}}{C_{\text{irr}} + C_{\text{non-irr}}}\,. \tag{13}$$

3 Uncertainty analysis

Food belief

Table S3 lists the datasets and the distributions defined to characterize the uncertainty in Equation 11 in the main manuscript.

Table S3: Variables used in the food belief uncertainty analysis. $\mathcal{TN}(\mu, \delta, a, b)$ stands for truncated normal distribution, where a and b denote the minimum and the maximum value of the population sample. Distributions for average yield of irrigated and non-irrigated grain are defined after bootstrapping with replacement ($N = 10^4$) the mean value of the aggregated data produced by the studies listed in the "Source column". * = distribution for wheat. † = distribution for maize.

Variable	Notation	Distribution	Unit	Source
Area of irrigated grain land	a_{irr}	$\mathcal{U}(296, 398)$	Mha	[1, 17, 25–28]
Area of non-irrigated grain land	$a_{\text{non-irr}}$	$\mathcal{U}(701, 1603)$	Mha	[29, 30]
Average yield of irrigated grain	y_{irr}			
		$\mathcal{TN}(4.3, 0.17, 3.68, 5.14)^*$	Tons/hectare	[13, 31–34]
		$\mathcal{TN}(7.18, 0.43, 5.42, 8.93)^\dagger$	Tons/hectare	[13, 32–34]
Average yield of non-irrigated grain	$y_{\text{non-irr}}$			
		$\mathcal{TN}(2.83, 0.13, 2.36, 3.42)^*$	Tons/hectare	[13, 31–34]
		$\mathcal{TN}(4.67, 0.36, 3.21, 6.12)^\dagger$	Tons/hectare	[13, 32–34]

We also explored the uncertainty in the 40% figure when the focus is placed not on production but on calories (the Rosa et al. [24] approach, Equation 13 in the Supplementary Materials). We used the values reported by FAOSTAT [35] for all wheat and maize-derived products (e.g., flour of maize/wheat, starch of wheat, germ of maize) to characterize the uncertainty in c_i, and denoted $c_{\text{wheat}} \sim \mathcal{U}(213, 382)$ and $c_{\text{maize}} \sim \mathcal{U}(356, 380)$. The results are shown in Figs. S8–S9.

Water belief

We describe the uncertainty in industrial, domestic and irrigation water withdrawals at the country level as $\mathcal{U}(\min_j, \max_j)$, where $\min_j, \max_j$ are the minimum and maximum values for country j given the data available. We retrieved irrigation water withdrawal (IWW) values from Puy et al. [36] and Puy [37], who showed that a regression against the extension of irrigation, combined with an uncertainty analysis, yields IWW estimates largely spanning the range of values produced by eight global hydrological models (WaterGap, VIC, MPI-HM, PCR-GLOBWB, DBHM, H08, CLM4.5, LPJmL) and two FAO-based datasets [1, 38]. For industrial and domestic water withdrawals, we retrieved 2010 estimates from seven sources that range from statistical and mixed-approaches to fully model-based exercises:

1. Aquastat [1]: annual data from country surveys, questionnaires, literature reviews and collaboration with agencies.
2. Gleick [39]: annual FAO data including estimated, measured, modelled or derived data. This is the only dataset in our IWW corpus with data before 2010 (∼1970-1995).
3. Flörke et al. [40]: data from 1950–2010 produced by the model WaterGAP3 on domestic and industrial (manufacturing + electricity) water withdrawals.
4. Liu et al. [38]: Aquastat-based data with missing values filled using inverse distance weighting, nearest neighbor or linear interpolations).
5. Khan et al. [41]: monthly sectoral water use (2010–2100) from the Global Change Analysis Model (GCAM4) across four Representation Concentration Pathways (RCPs), five Shared Socioeconomic Pathways (SSPs) and five Global Climate Models].
6. Huang et al. [42, 43]: monthly sectoral water use (1971–2010) from downscaled Aquastat data combined with simulations from the Global Change Assessment Model].
7. The inter-sectoral impact model intercomparison project (ISI–MIP) [44]: simulations from MATSIRO, PCR-GLOBWB, H08, CWATM and MIROC with GSWP3 and ESM4 climate forcing.

Since the data from 5) to 7) are produced at a spatial resolution of $0.5^{\circ} \times 0.5^{\circ}$, we allocated each cell to a specific country given its geospatial information (longitude and latitude) and produced annual irrigation, industrial and domestic water withdrawal values (Km^3) by adding the values of all cells in the same country after correcting for the grid cell size.

4 Figures

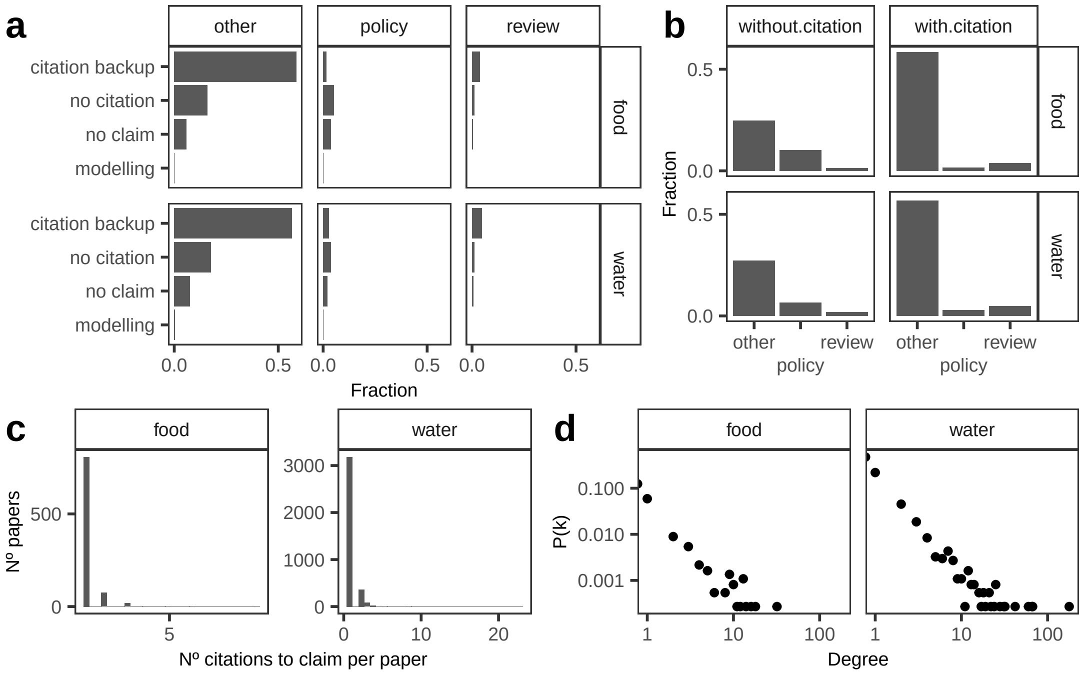

Figure S1: Descriptive statistics. a) Proportion of document types (top facets) as a function of the nature of their claim (x-axis). b) Proportion of document types with or without a citation to support the claim. c) Histogram showing the distribution of the number of citations to the claim per paper. d) log-log plot displaying the probability of a node displaying a degree k. The trend is reminiscent of a power-law and a scale-free network.

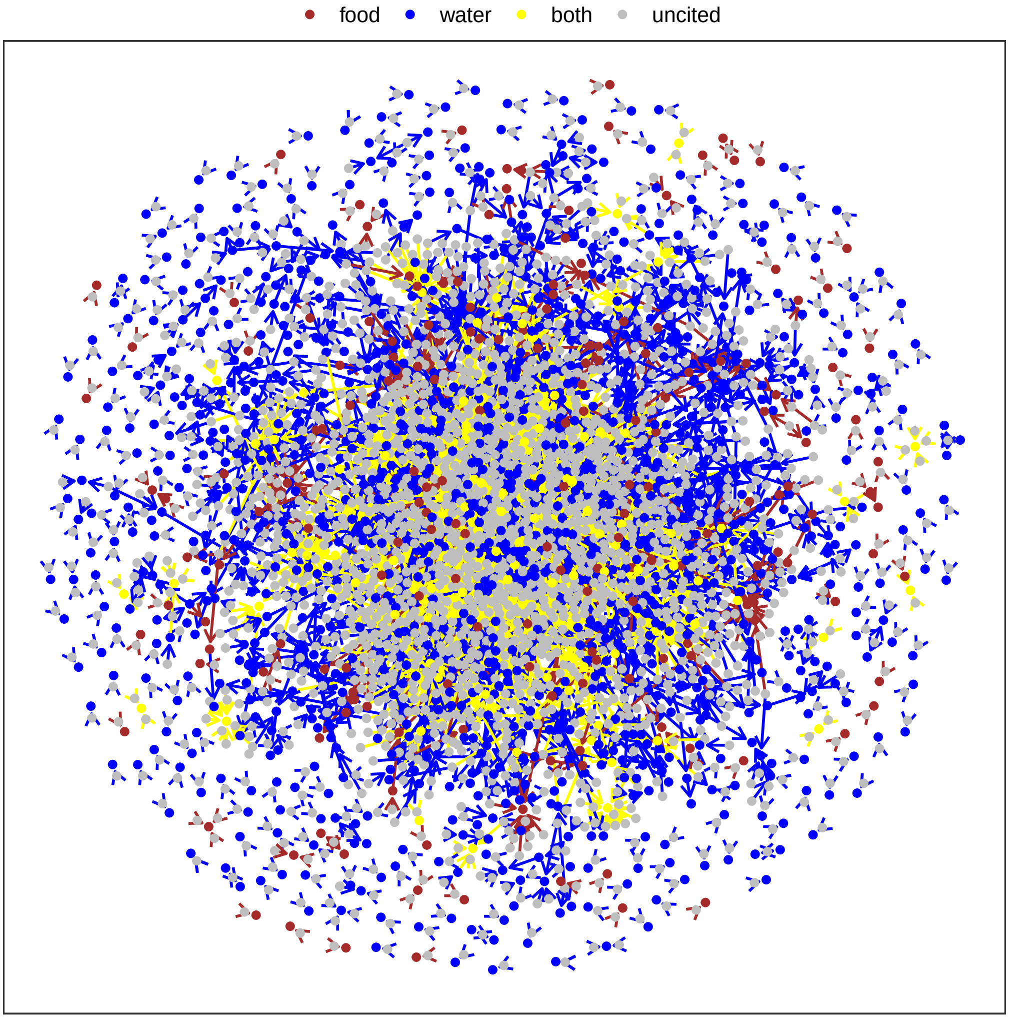

Figure S2: Full network including the food and the water belief systems. The yellow dots denote documents that are cited as sources for both claims. The grey dots show documents that make either claim and cite another document to support it but are not cited by other documents in the network.

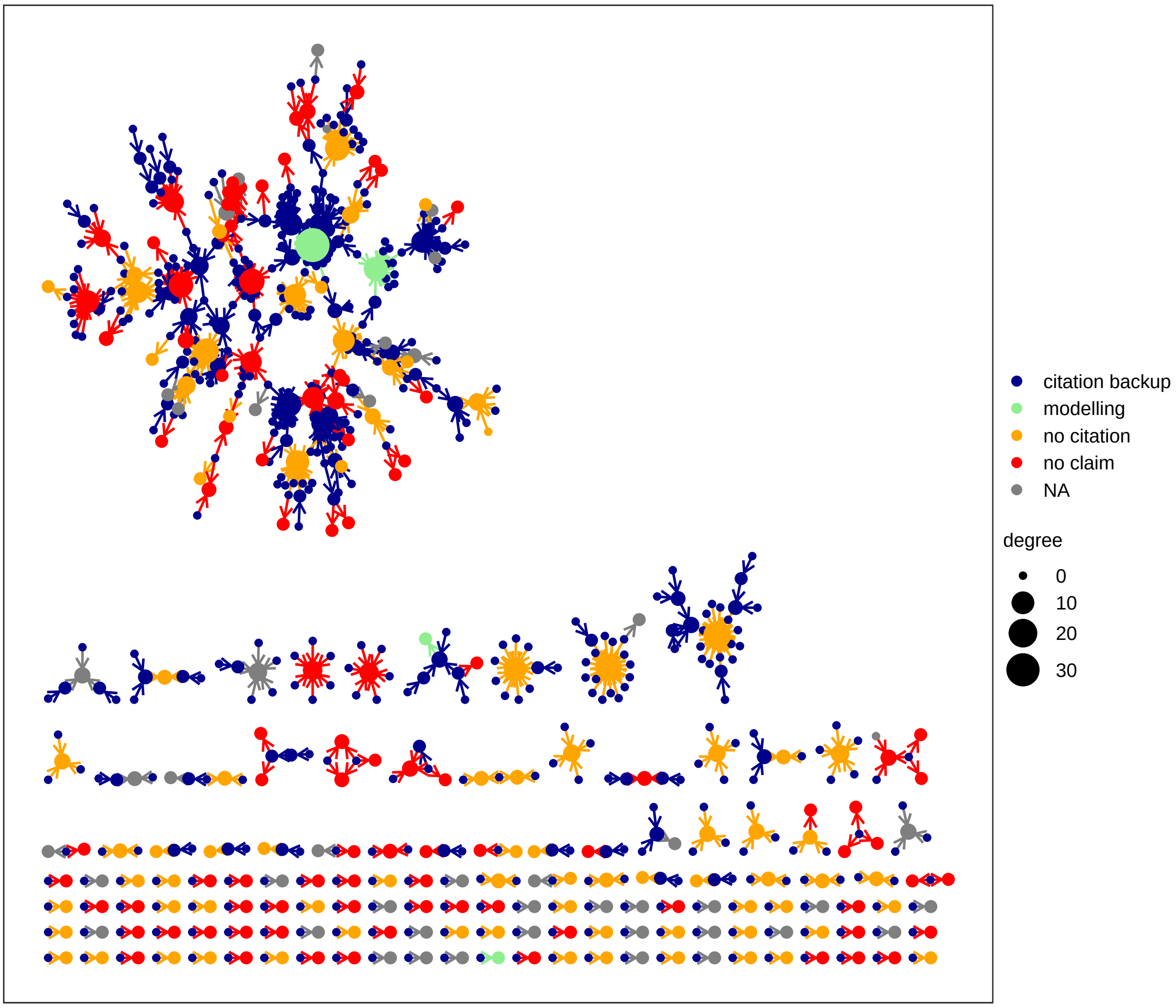

Figure S3: Food belief network. The size of the nodes (documents) correspond to their degree; that is, to the number of incoming edges (citations). Blue nodes ("citation backup") denote documents that make the 40% claim and support the claim with a citation. Green nodes ("modelling") represent documents that produce original data supporting the claim through a modelling or statistical exercise. Orange nodes ("no citation") are documents that make the claim but do not produce original data nor cite any study to support the claim. Red nodes ("no claim") are documents that are cited to support the claim but do not actually make the claim. Grey nodes ("NA") represent documents that are cited as making the claim but that we have been unable to access.

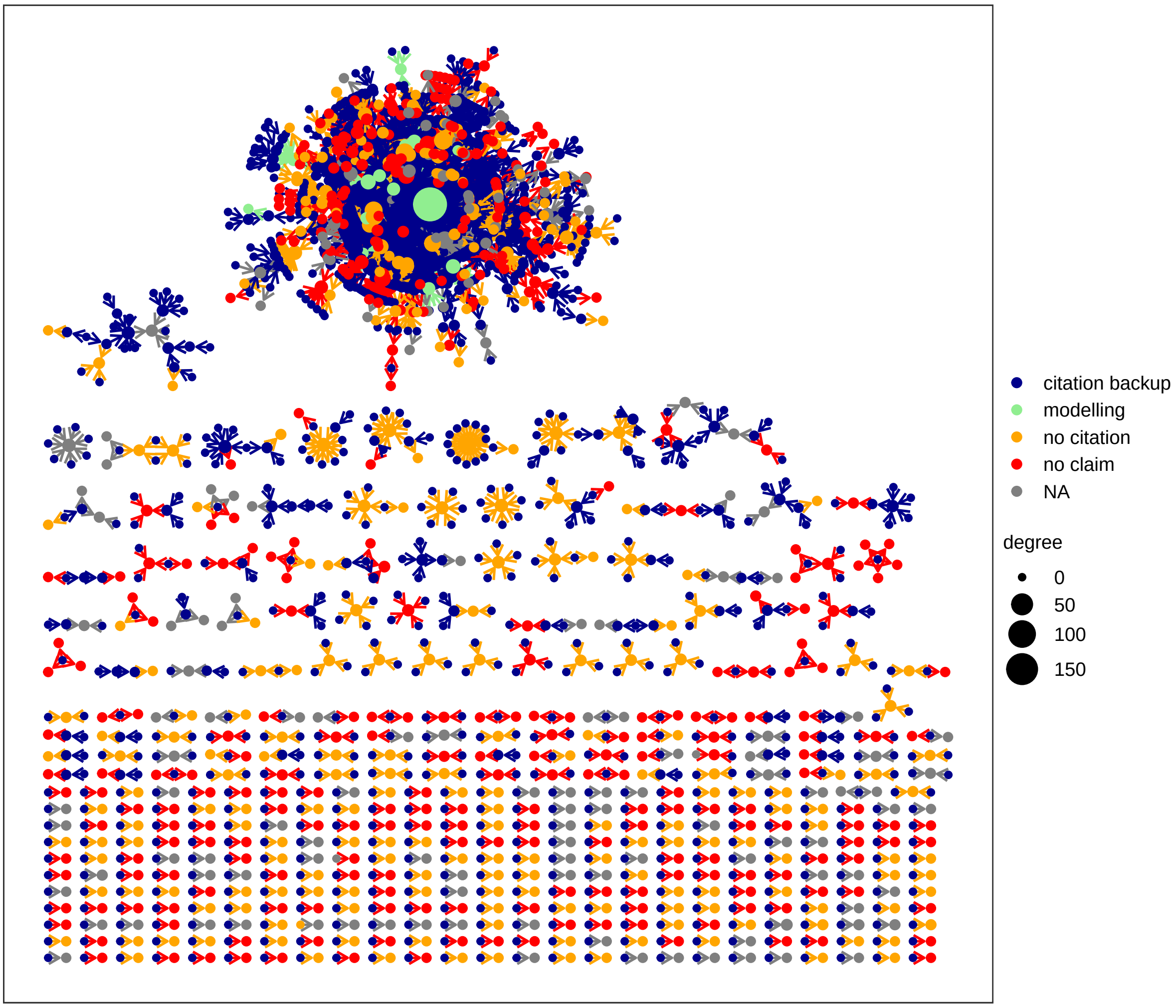

Figure S4: Water belief network. The size of the nodes (documents) correspond to their degree; that is, to the number of incoming edges (citations). Blue nodes ("citation backup") denote documents that make the 70% claim and support the claim with a citation. Green nodes ("modelling") represent documents that produce original data supporting the claim through a modelling or statistical exercise. Orange nodes ("no citation") are documents that make the claim but do not produce original data nor cite any study to support the claim. Red nodes ("no claim") are documents that are cited to support the claim but do not actually make the claim. Grey nodes ("NA") represent documents that are cited as making the claim but that we have been unable to access.

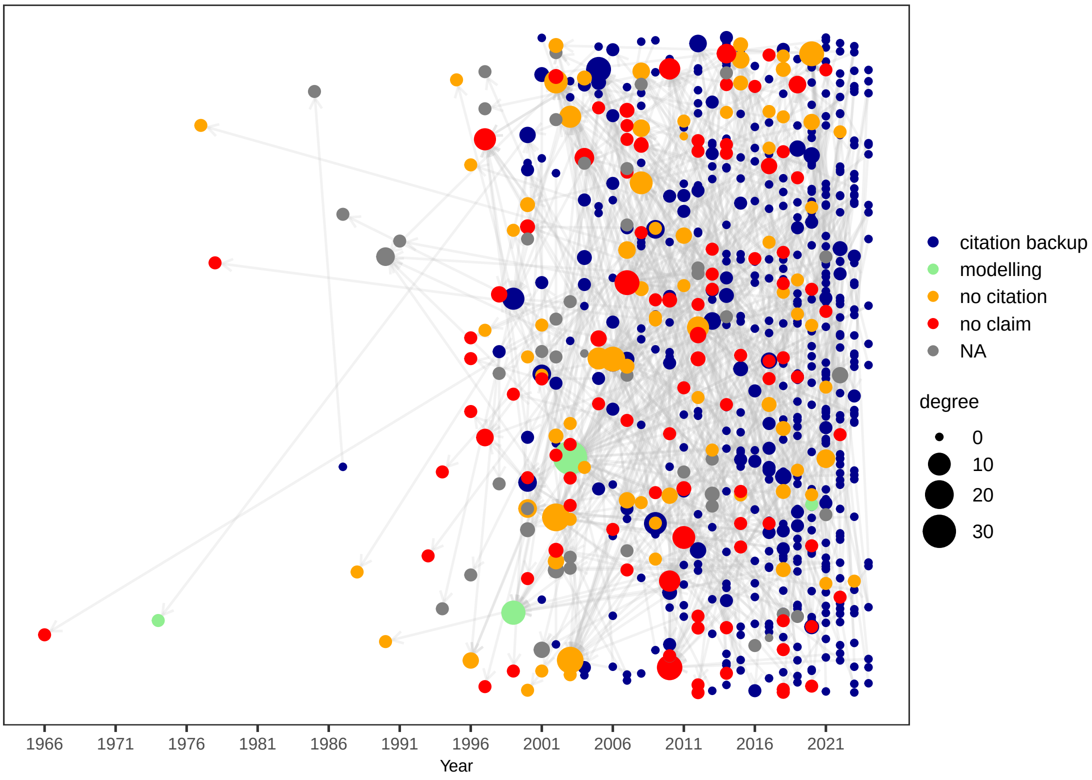

Figure S5: Food belief network as a function of time (x-axis).

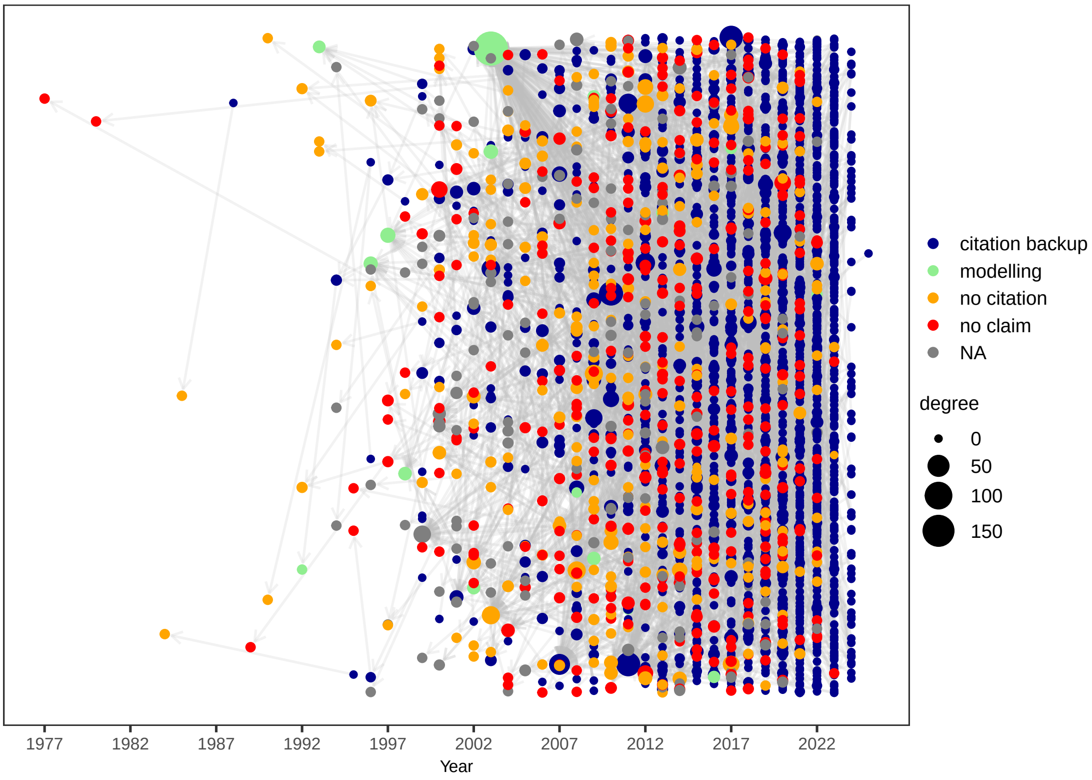

Figure S6: Water belief network as a function of time (x-axis).

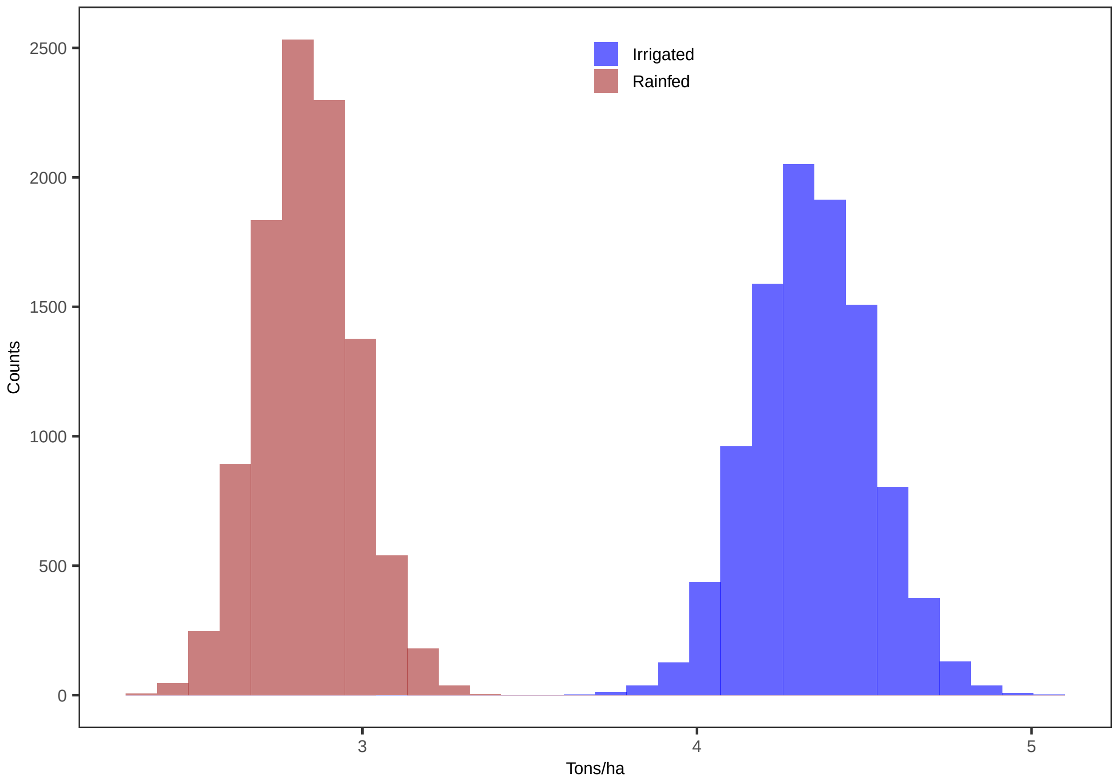

Figure S7: Uncertainty in the average production of wheat in irrigated and in rainfed areas after bootstrapping with replacement the mean value of the data compiled in Table S3.

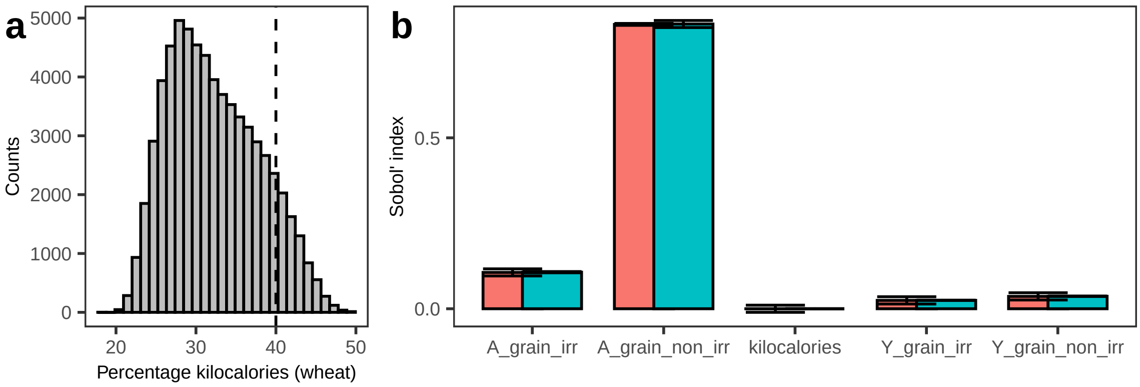

Figure S8: Uncertainty and sensitivity analysis of the percentage of calories derived from irrigated wheat. a) Uncertainty analysis. b) Sobol' indices. See the main manuscript for a description of S_i and T_i.

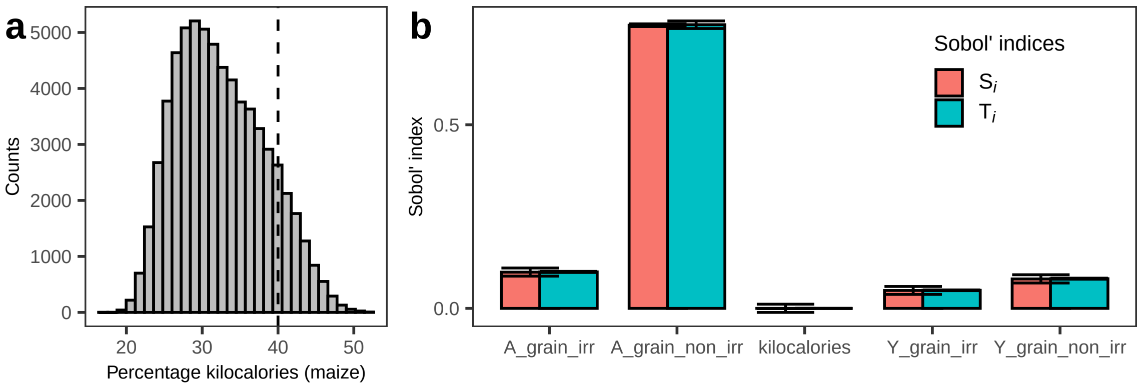

Figure S9: Uncertainty and sensitivity analysis of the percentage of calories derived from irrigated maize. a) Uncertainty analysis. b) Sobol' indices. See the main manuscript for a description of S_i and T_i.

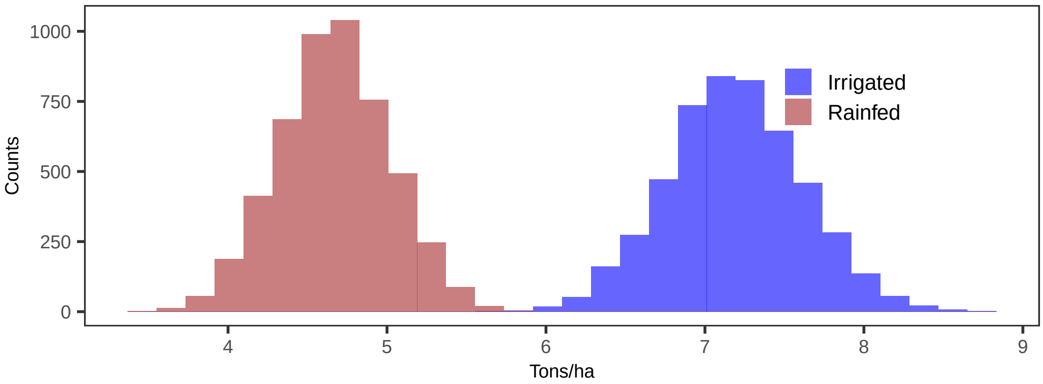

Figure S10: Uncertainty in the average production of maize in irrigated and in rainfed areas after bootstrapping with replacement the mean value of the data compiled in Table S3.

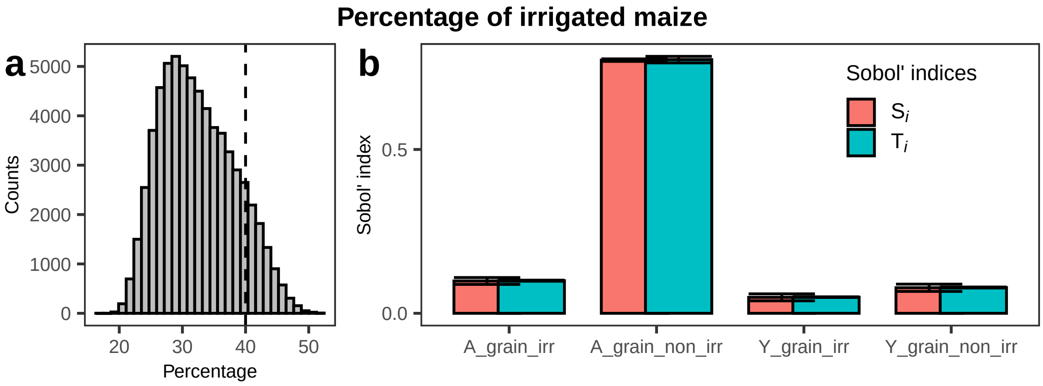

Figure S11: Uncertainty and sensitivity analysis of the percentage of maize produced in irrigated areas. a) Monte Carlo-based uncertainty analysis. b) Sobol' indices. See the main manuscript for a description of S_i and T_i.

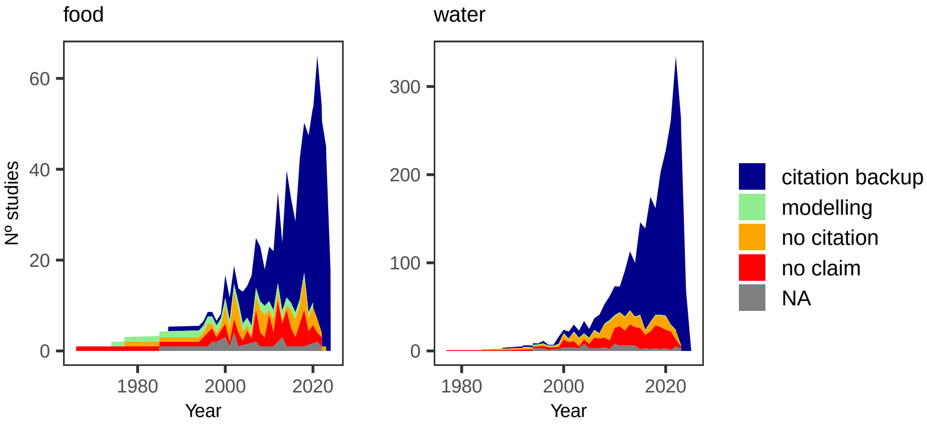

Figure S12: Number of studies in the food and the water belief systems over time.

- **Classification**
  - 1: Document type
    - Review
    - Policy
    - Other (book chapter, journal paper, book)
  - 2: Nature of claim:
    - Primary
      - Modelling
    - Secondary
      - Citation backup
      - No citation
    - No claim
  - 3: Citation/s supporting the claim (if nature of claim is "citation backup")
  - 4: Strength of claim
    - Fact
    - Approximate
    - Upper
    - Lower
    - Range

Figure S13: Tree diagram showing the classification scheme.

References

[1] *AQUASTAT - FAO's Global Information System on Water and Agriculture*. 2024. URL: https://www.fao.org/aquastat/en/overview/ (visited on 10/04/2024).

[2] S. Siebert, J. Burke, J. M. Faures, K. Frenken, J. Hoogeveen, P. Döll, and F. T. Portmann. "Groundwater Use for Irrigation – a Global Inventory". *Hydrology and Earth System Sciences* 14.10 (Oct. 12, 2010), 1863–1880. DOI: 10.5194/hess-14-1863-2010.

[3] Food And Agriculture Organization Of The United Nations. *The State of the World's Land and Water Resources for Food and Agriculture. Managing Systems at Risk*. 0th ed. Routledge, 2011. DOI: 10.4324/9780203142837.

[4] D. Molden, ed. *Water for Food, Water for Life: A Comprehensive Assessment of Water Management in Agriculture*. London: Earthscan, 2007. 645 pp.

[5] Food And Agriculture Organization Of The United Nations. *Water for Sustainable Food and Agriculture. A Report Produced for the G20 Presidency of Germany*. Rome: FAO, 2017.

[6] World Water Assessment Programme, ed. *The United Nations World Water Development Report 4. Managing Water Under Uncertainty and Risk*. The United Nations World Water Development Report 4. Paris: UNESCO publ, 2012.

[7] J. A. Foley, N. Ramankutty, K. A. Brauman, E. S. Cassidy, J. S. Gerber, M. Johnston, N. D. Mueller, C. O'Connell, D. K. Ray, P. C. West, C. Balzer, E. M. Bennett, S. R. Carpenter, J. Hill, C. Monfreda, S. Polasky, J. Rockström, J. Sheehan, S. Siebert, D. Tilman, and D. P. M. Zaks. "Solutions for a Cultivated Planet". *Nature* 478.7369 (Oct. 2011), 337–342. DOI: 10.1038/nature10452.

[8] United Nations. *Water for People, Water for Life. The United Nations World Water Development Report*. United Nations, 2003.

[9] M. W. Rosegrant, C. Ringler, and T. Zhu. "Water for Agriculture: Maintaining Food Security under Growing Scarcity". *Annual Review of Environment and Resources* 34 (Volume 34, 2009 Nov. 21, 2009), 205–222. DOI: 10.1146/annurev.environ.030308.090351.

[10] S. Rost, D. Gerten, A. Bondeau, W. Lucht, J. Rohwer, and S. Schaphoff. "Agricultural Green and Blue Water Consumption and Its Influence on the Global Water System". *Water Resources Research* 44.9 (Sept. 2008), 2007WR006331. DOI: 10.1029/2007WR006331.

[11] Food And Agriculture Organization Of The United Nations. *Crops and Drops. Making the Best Use of Water for Agriculture*. Rome: Food and Agriculture Organization of the United Nations, 2002.

[12] B. L. Morris, A. R. L. Lawrence, P. J. C. Chilton, B. Adams, R. C. Calow, and B. A. Klinck. *Groundwater and Its Susceptibility to Degradation : A Global Assessment of the Problem and Options for Management*. Vol. 03–3. United Nations Environment Programme, 2003. 126 pp.

[13] S. Siebert and P. Döll. "Quantifying Blue and Green Virtual Water Contents in Global Crop Production as Well as Potential Production Losses without Irrigation". *Journal of Hydrology* 384.3-4 (2010), 198–217. DOI: 10.1016/j.jhydrol.2009.07.031.

[14] S. Siebert, P. Döll, J. Hoogeveen, J.-M. Faures, K. Frenken, and S. Feick. "Development and Validation of the Global Map of Irrigation Areas". *Hydrology and Earth System Sciences* 9.5 (Nov. 16, 2005), 535–547. DOI: 10.5194/hess-9-535-2005.

[15] World Bank. *Water in Agriculture*. World Bank. 2020. URL: https://www.worldbank.org/en/news/infographic/2023/07/26/water-in-agriculture (visited on 10/04/2024).

[16] K. bin Abdullah. "Use of Water and Land for Food Security and Environmental Sustainability". *Irrigation and Drainage* 55.3 (2006), 219–222. DOI: 10.1002/ird.254.

[17] Food And Agriculture Organization Of The United Nations. *FAOSTAT*. 2024. URL: https://www.fao.org/faostat/en/#home (visited on 10/04/2024).

[18] P. Döll and S. Siebert. "Global Modeling of Irrigation Water Requirements". *Water Resources Research* 38.4 (2002), 8–1–8–10. DOI: 10.1029/2001WR000355. pmid: 11141563.

[19] M. I. L'vovich. *World Water Resources and their Future*. Translated from the Russian Edition (1974) by the American Geophysical Union. Moscow: American Geophysical Union, 1979.

[20] J. A. Parkinson. *Irrigation in the Near East Region in figures*. Fao Rome, Italy, 1997.

[21] I. Oudra, D. Niriella, K. Frenken, and M. Bousquet. "Irrigation in Asia in figures". *Water Reports (FAO)* 18 (1999).

[22] F. Karen. *Irrigation in the Middle East region in figures*. 2009.

[23] K. Frenken et al. "Irrigation in Southern and Eastern Asia in figures" (2011).

[24] L. Rosa, D. D. Chiarelli, M. C. Rulli, J. Dell'Angelo, and P. D'Odorico. "Global Agricultural Economic Water Scarcity". *Science Advances* 6.18 (May 2020), eaaz6031. DOI: 10.1126/sciadv.aaz6031.

[25] S. Siebert, P. Döll, J. Hoogeveen, J.-M. Faures, K. Frenken, and S. Feick. "Development and Validation of the Global Map of Irrigation Areas". *Hydrology and Earth System Sciences* 9.5 (Nov. 2005), 535–547. DOI: 10.5194/hess-9-535-2005.

[26] J. Meier, F. Zabel, and W. Mauser. "A Global Approach to Estimate Irrigated Areas. A Comparison between Different Data and Statistics". *Hydrology and Earth System Sciences* 22.2 (2018), 1119–1133. DOI: 10.5194/hess-22-1119-2018.

[27] P. S. Thenkabail, C. M. Biradar, H. Turral, P. Noojipady, Y. J. Li, J. Vithanage, V. Dheeravath, M. Velpuri, M. Schull, X. L. Cai, and R. Dutta. *An Irrigated Area Map of the World (1999) Derived from Remote Sensing*. June 2014. Colombo: International Water Management Institute, 2006.

[28] J. Salmon, M. A. Friedl, S. Frolking, D. Wisser, and E. M. Douglas. "Global Rain-Fed, Irrigated, and Paddy Croplands: A New High Resolution Map Derived from Remote Sensing, Crop Inventories and Climate Data". *International Journal of Applied Earth Observation and Geoinformation* 38 (2015), 321–334. DOI: 10.1016/j.jag.2015.01.014.

[29] F. N. Tubiello, G. Conchedda, L. Casse, P. Hao, G. De Santis, and Z. Chen. "A New Cropland Area Database by Country circa 2020". *Earth System Science Data* 15.11 (Nov. 15, 2023), 4997–5015. DOI: 10.5194/essd-15-4997-2023.

[30] P. Potapov, S. Turubanova, M. C. Hansen, A. Tyukavina, V. Zalles, A. Khan, X.-P. Song, A. Pickens, Q. Shen, and J. Cortez. "Global Maps of Cropland Extent and Change Show Accelerated Cropland Expansion in the Twenty-First Century". *Nature Food* 3.1 (Dec. 23, 2021), 19–28. DOI: 10.1038/s43016-021-00429-z.

[31] A. Dadrasi, M. Chaichi, A. Nehbandani, E. Soltani, A. Nemati, F. Salmani, M. Heydari, and A. R. Yousefi. "Global insight into understanding wheat yield and production through Agro-Ecological Zoning". *Scientific Reports* 13.1 (Sept. 23, 2023), 15898. DOI: 10.1038/s41598-023-43191-x.

[32] Food And Agriculture Organization Of The United Nations. *GAEZ Data Portal. Crop Summary. Global Crop Profile and Crop Statistics*. 2024. URL: https://gaez.fao.org/pages/crop-summary (visited on 10/28/2024).

[33] N. D. Mueller, J. S. Gerber, M. Johnston, D. K. Ray, N. Ramankutty, and J. A. Foley. "Closing Yield Gaps through Nutrient and Water Management". *Nature* 490.7419 (Oct. 11, 2012), 254–257. DOI: 10.1038/nature11420.

[34] International Food Policy Research Institute (IFPRI). *Global Spatially-Disaggregated Crop Production Statistics Data for 2020 Version 1.0*. Version 3.0. Harvard Dataverse, 2024. DOI: 10.7910/DVN/SWPENT.

[35] FAOSTAT. *Food Balance Sheets - A Handbook*. URL: https://www.fao.org/4/X9892E/X9892e05.htm#P8217_125315 (visited on 11/22/2024).

[36] A. Puy, E. Borgonovo, S. Lo Piano, S. A. Levin, and A. Saltelli. "Irrigated Areas Drive Irrigation Water Withdrawals". *Nature Communications* 12.1 (Dec. 2021), 4525. DOI: 10.1038/s41467-021-24508-8.

[37] A. Puy. *R Code of the Paper "Irrigated Areas Drive Irrigation Water Withdrawals"*. Version 2.0.1. Zenodo, Apr. 2021. DOI: 10.5281/zenodo.4721393.

[38] Y. Liu, M. Hejazi, P. Kyle, S. H. Kim, E. Davies, D. G. Miralles, A. J. Teuling, Y. He, and D. Niyogi. "Global and Regional Evaluation of Energy for Water". *Environmental Science and Technology* 50.17 (2016), 9736–9745. DOI: 10.1021/acs.est.6b01065.

[39] P. H. Gleick. *The World's Water, 2000-2001: The Biennial Report on Freshwater Resources*. Washington, D.C: Island Press, 2000. 316 pp.

[40] M. Flörke, E. Kynast, I. Bärlund, S. Eisner, F. Wimmer, and J. Alcamo. "Domestic and Industrial Water Uses of the Past 60 Years as a Mirror of Socio-Economic Development: A Global Simulation Study". *Global Environmental Change* 23.1 (Feb. 1, 2013), 144–156. DOI: 10.1016/j.gloenvcha.2012.10.018.

[41] Z. Khan, I. Thompson, C. R. Vernon, N. T. Graham, T. B. Wild, and M. Chen. "Global Monthly Sectoral Water Use for 2010–2100 at 0.5° Resolution across Alternative Futures". *Scientific Data* 10.1 (Apr. 11, 2023), 201. DOI: 10.1038/s41597-023-02086-2.

[42] Z. Huang, M. Hejazi, X. Li, Q. Tang, C. Vernon, G. Leng, Y. Liu, P. Döll, S. Eisner, D. Gerten, N. Hanasaki, and Y. Wada. *Global Gridded Monthly Sectoral Water Use Dataset for 1971-2010: V2*. 2018. DOI: 10.5281/ZENODO.1209296.

[43] Z. Huang, M. Hejazi, X. Li, Q. Tang, C. Vernon, G. Leng, Y. Liu, P. Döll, S. Eisner, D. Gerten, N. Hanasaki, and Y. Wada. "Reconstruction of Global Gridded Monthly Sectoral Water Withdrawals for 1971–2010 and Analysis of Their Spatiotemporal Patterns". *Hydrology and Earth System Sciences* 22.4 (Apr. 6, 2018), 2117–2133. DOI: 10.5194/hess-22-2117-2018.

[44] L. Warszawski, K. Frieler, V. Huber, F. Piontek, O. Serdeczny, and J. Schewe. "The Inter-Sectoral Impact Model Intercomparison Project (ISI–MIP): Project Framework". *Proceedings of the National Academy of Sciences* 111.9 (Mar. 2014), 3228–3232. DOI: 10.1073/pnas.1312330110.
